# Induced inactivation of *Wnt16* in young adult mice has no impact on osteoarthritis development

**DOI:** 10.1371/journal.pone.0277495

**Published:** 2022-11-11

**Authors:** Anna E. Törnqvist, Karin H. Nilsson, Lei Li, Claes Ohlsson, Sofia Movérare-Skrtic

**Affiliations:** 1 Sahlgrenska Osteoporosis Centre, Centre for Bone and Arthritis Research, Institute of Medicine, Sahlgrenska Academy at University of Gothenburg, Gothenburg, Sweden; 2 Region Västra Götaland, Department of Drug Treatment, Sahlgrenska University Hospital, Gothenburg, Sweden; Medical College of Wisconsin, UNITED STATES

## Abstract

Osteoarthritis (OA) is a common disorder and a major cause of disability in the elderly population. WNT16 has been suggested to play important roles in joint formation, bone homeostasis and OA development, but the mechanism of action is not clear. Transgenic mice lacking *Wnt16* expression (*Wnt16*^*-/-*^) have a more severe experimental OA than control mice. In addition, *Wnt16*^*-/-*^ mice have a reduced cortical thickness and develop spontaneous fractures. Herein, we have used *Cre-Wnt16*^*flox/flox*^ mice in which *Wnt16* can be conditionally ablated at any age through tamoxifen-inducible Cre-mediated recombination. *Wnt16* deletion was induced in 7-week-old mice to study if the *Cre-Wnt16*^*flox/flox*^ mice have a more severe OA phenotype after destabilizing the medial meniscus (DMM surgery) than littermate controls with normal *Wnt16* expression (*Wnt16*^*flox/flox*^). WNT16 deletion was confirmed in articular cartilage and cortical bone in *Cre-Wnt16*^*flox/flox*^ mice, shown by immunohistochemistry and reduced cortical bone area compared to *Wnt16*^*flox/flox*^ mice. After DMM surgery, there was no difference in OA severity in the articular cartilage in the knee joint between the *Cre-Wnt16*^*flox/flox*^ and *Wnt16*^*flox/flox*^ mice in neither female nor male mice. In addition, there was no difference in osteophyte size in the DMM-operated tibia between the genotypes. In conclusion, inactivation of *Wnt16* in adult mice do not result in a more severe OA phenotype after DMM surgery. Thus, presence of WNT16 in adult mice does not have an impact on experimental OA development. Taken together, our results from *Cre-Wnt16*^*flox/flox*^ mice and previous results from *Wnt16*^*-/-*^ mice suggest that WNT16 is crucial during synovial joint establishment leading to limited joint degradation also later in life, after onset of OA. This may be important when developing new therapeutics for OA treatment.

## Introduction

Osteoarthritis (OA) is a common disorder and a major cause of disability in the elderly population. It is a disease affecting the whole joint typically characterized by alterations of the articular cartilage, abnormal subchondral bone remodeling with osteophyte formation, and structural alterations in the synovial membrane, ligaments, and capsule [1, 2]. The pathogenesis of OA is complex and largely unknown, but several lines of evidence suggest involvement of WNT signaling [3, 4]. WNT activity is essential for both cartilage and bone development and maintenance [5]. Polymorphisms in the *SFRP3* gene, which encodes Frizzled-related protein (FRZB), a secreted antagonist of the WNT signaling cascade, was found to be associated with hip osteoarthritis already in 2004 [6], and mice lacking FRZB have increased susceptibility to OA [7, 8]. Cartilage-specific inactivation of beta-catenin, a key molecule for WNT signaling, leads to articular cartilage destruction in mice [9], whereas cartilage-specific overexpression of a constitutively active form of beta-catenin also results in OA due to increased cartilage cell hypertrophy [10]. Furthermore, activation of WNT signaling is observed in humans in both articular cartilage and synovium following injury, with increased expression of both WNT ligands and target genes [11, 12]. Thus, the WNT signaling pathway is considered a potential target for OA treatment.

One of the WNT proteins that has gained more interest during the last years is WNT16, which has been linked to bone mass and is suggested to have a protective role during OA development [12–17]. The WNT16 locus has been identified as a major determinant of cortical thickness, forearm bone mineral density (BMD), and fractures in humans [13, 17]. Subsequent studies in mice with global deletion of *Wnt16* (*Wnt16*^*-/-*^ mice) demonstrated thinner cortical thickness and spontaneous fractures [14]. WNT16 was upregulated both in human OA cartilage [12, 16], and after mechanic injury to human articular cartilage in an ex vivo model [12]. It has also been demonstrated that *Wnt16*^*-/-*^ mice developed a more severe OA phenotype than WT controls after inducing OA by destabilizing the medial meniscus (DMM) [15]. In addition, mice with a chondrocyte specific deletion of *Wnt16* (named *Wnt16-cKO* by *Tong et al*) also displayed a more severe OA phenotype after anterior cruciate ligament transection (ACLT) surgery compared to WT controls [16]. Furthermore, injection of an adenoviral vector overexpressing *Wnt16* into either a WT or chondrocyte specific *Wnt16-KO* mouse knee with experimentally induced OA attenuated the OA phenotype [16]. Thus, WNT16 is suggested to protect articular cartilage from damage in experimental OA models. However, if an adenoviral vector overexpressing *Wnt16* was injected into a healthy WT mouse knee it caused damage to the articular cartilage [18]. This suggests that WNT16 is important for protecting the articular cartilage during an ongoing OA development but exerts negative effects on healthy undamaged cartilage.

The mouse models with global *Wnt16* deletion or conditional *Wnt16* deletion in chondrocytes both show that WNT16 is important for protecting the articular cartilage from experimental OA in mice [15, 16]. However, as the global *Wnt16*^*-/-*^ and the chondrocyte specific *Wnt16-KO* mouse models were born with the *Wnt16* deletion and since WNT16 has been shown to play a role in synovial joint establishment during embryonic development [19, 20], it is possible that these mice developed a more severe experimental OA due to the lack of WNT16 during development. In order to separate the developmental effects of WNT16 from the effects of WNT16 in the adult mice on OA progression, we have developed a mouse model in which *Wnt16* can be conditionally ablated through tamoxifen-inducible Cre-mediated recombination (*Cre-Wnt16*^*flox/flox*^ mice) [21]. These mice express *Wnt16* during development and growth, and can be exposed to tamoxifen to induce *Wnt16* deletion at any age. We have previously shown that adult *Cre-Wnt16*^*flox/flox*^ mice develop a reduced cortical thickness after deleting *Wnt16* [21]. Herein, using the previously developed tamoxifen-inducible *Wnt16* deleted mouse model, we investigated if the *Cre-Wnt16*^*flox/flox*^ mice, after deletion of *Wnt16* expression in adult mice, develop a more severe experimental OA after DMM surgery than littermate controls with normal *Wnt16* expression (*Wnt16*^*flox/flox*^).

## Materials and methods

### Animals

We have recently described the generation of tamoxifen-inducible *Wnt16* knockout mice [21]. Briefly, B6.Cg-Tg(CAG-cre/Esr1*)5Amc/J (CAGGCre-ER) transgenic mice expressing a tamoxifen-inducible Cre-mediated recombination system (JAX stock #004682) [22] were used and mated with *Wnt16*^*flox/flox*^ mice on a C57BL/6N background. The *Wnt16*^*flox/flox*^ mice have *loxP* sites flanking exon 3 of the *Wnt16* gene. The tamoxifen-inducible offspring, CAGGCre-ER-*Wnt16*^*flox/flox*^ mice, are referred to as *Cre-Wnt16*^*flox/flox*^. The control mice were littermate *Wnt16*^*flox/flox*^ mice without CAGGCre-ER expression. All experiments were carried out in both female and male mice. The mice were housed together in a standard animal facility under controlled temperature (22°C) and photo periods (12 h of light, 12 h of dark) with free access to water and food pellets (RM1A, SDS Diet, UK) Animal care was in accordance with institutional guidelines. All applicable international, national, and institutional guidelines for the care and use of animals were followed. All procedures performed were approved by the Ethics Committee in Gothenburg, Västra Götaland (Permit Number 79–2015), and the care of the animals was in compliance with all relevant ethical regulations for animal testing and research.

### Tamoxifen treatment

Tamoxifen (T5648, Sigma-Aldrich) was dissolved in ethanol at a concentration of 100 mg/mL and further diluted in corn oil (C8267, Sigma-Aldrich) to a concentration of 10 mg/mL. The tamoxifen suspension was administered to the *Cre-Wnt16*^*flox/flox*^ mice and their *Wnt16*^*flox/flox*^ littermates by intraperitoneal injections for four consecutive days. This will delete the floxed exon 3 of the *Wnt16* gene in the mice with tamoxifen-inducible Cre recombinase (the *Cre-Wnt16*^*flox/flox*^ mice) [21]. Seven-week-old female and male mice were given 50 mg/kg tamoxifen, which corresponds to the dose previously used [21].

### Surgically induced osteoarthritis

Experimental OA was induced in 8-week-old male and female *Cre-Wnt16*^flox/flox^ and *Wnt16*^*flox/flox*^ mice. The surgery was performed 4 days after the last tamoxifen injection in females and 5 days after the last tamoxifen injection in males. This was done by surgically destabilizing the medial meniscus (DMM) in the right knee essentially as described by Glasson et al [23]. In brief, the fur surrounding the knee joints was shaved and a skin incision was made over the medial aspect of the knee joint under inhalation anesthesia with Isoflurane (Forene; Abbot Scandinavia, Solna, Sweden). The joint cavity was opened, the medial meniscus and the medial meniscotibial ligament were identified, and thereafter the medial meniscotibial ligament was sectioned using a microsurgical knife. Care was taken to avoid damaging other ligaments and cartilage. The skin was then sutured (DMM females: *Cre-Wnt16*^*flox/flox*^ N = 12 and *Wnt16*^*flox/flox*^ N = 10; DMM males: *Cre-Wnt16*^*flox/flox*^ and *Wnt16*^*flox/flox*^ N = 11). Another group of mice was not subjected to DMM surgery, and the right knees of these mice were left un-operated and used as controls; referred to as controls (control females: *Cre-Wnt16*^*flox/flox*^ N = 12, *Wnt16*^*flox/flox*^ N = 10; control males: *Cre-Wnt16*^*flox/flox*^ and *Wnt16*^*flox/flox*^ N = 11). The mice undergoing DMM surgery received the analgesic Bupaq vet (0.3 mg/ml, Salfarm Scandinavia, Helsingborg, Sweden) at the time of surgery. The mice were carefully assessed for adverse events the first three days after surgery and then every week throughout the experiment. The mice were kept for 8 weeks post-operatively and sacrificed at 16 weeks of age. At the time of termination, the mice were anesthetized with intraperitoneal injection with ketamine and dexmedetomidine before cervical dislocation. The lower limbs were dissected and skin was removed. The limbs were fixed for 48 hours in 4% formaldehyde and then kept in ethanol (70% v/v) until further use.

#### Histological analysis

Histological analyses were performed on fixed, decalcified whole knee joints collected at the end of the experiment. The joints were then processed and embedded in paraffin wax according to standard techniques. Five μm coronal joint sections were collected at 80 μm intervals.

*Immunohistochemistry*. The sections were deparaffinized and rehydrated. Antigen retrieval was applied with Dako retrieval solution (S169984-2, Dako) followed by quenching of endogenous peroxidase activity with 3% H_2_O_2_. Primary antibody against WNT16 (sc-271897, Santa Cruz Biotechnology) was diluted 1:200 in blocking serum and incubated overnight at 4°C. After incubation, the sections were incubated again with a biotinylated secondary antibody (BA-9200-1.5, goat anti-mouse, Vectastain) for 1 hour and ABC-HRP Kit (PK-6100, Vectastain) for another 1 hour according to manufacturer’s instructions. Liquid DAB substrate chromogen system (K3468, Dako) was used as peroxidase substrate for enzymatic amplification and hematoxylin was applied for counterstain. After dehydration, slides were mounted in Pertex (00871.0500, Histolab).

*Analysis of OA*. The sections were stained with Toluidine blue according to standard techniques. Histopathologic evaluation of the severity of OA was performed by an observer (AET) blinded to genotype according to the Osteoarthritis Research Society International (OARSI) recommendation of a 0–6 subjective scoring system [24]. In this classification system 0 represents normal cartilage; 0.5: loss of Toluidine blue staining without structural changes; 1: small fibrillations without loss of cartilage; 2: vertical clefts below the superficial layer and some loss of surface lamina; 3: vertical clefts/erosion to the calcified cartilage extending to <25% of the articular surface; 4: vertical clefts/erosion to the calcified cartilage extending to 25-50% of the articular surface; 5: vertical clefts/erosion to the calcified cartilage extending to 50–75% of the articular surface; 6: vertical clefts/erosion to the calcified cartilage extending >75% of the articular surface [24]. The 0–6 subjective OA scoring system was applied to all four regions of the joint: the medial tibial plateau (MTP), medial femoral condyle (MFC), lateral tibial plateau (LTP) and lateral femoral condyle (LFC). The OA severity is expressed as the maximal OARSI score that was found in the sections for each of the four regions. The summed maximal OARSI scores for the total knee joint (MTP + MFC + LTP + LFC) was also used. The intra-class correlation coefficient (ICC) between OARSI scores for AET and a second observer is 0.940 (95% confidence interval: 0.926, 0.951), which is considered excellent [25].

The thickness of the total articular cartilage was measured using the OsteoMeasure histomorphometry system, software version 2.2 (OsteoMetric, Atlanta, GA) as described previously [26]. Ten measurements were taken evenly distributed across the measurement area of one section per mouse and the mean from the ten measurements was used for each mouse.

#### Analyses of cortical bone by peripheral quantitative computed tomography (pQCT)

Scans of the cortical mid-diaphyseal femur were performed using pQCT XCT Research M (version 4.5B; Norland, Fort Atkinson, USA) operating at a resolution of 70 μm as described previously [27, 28].

### Analysis of subchondral bone by micro CT (μCT)

Analysis of subchondral bone was performed by μCT using a Skyscan 1172 model micro-CT (Bruker micro-CT, Aartselaar, Belgium) [14] with an X-ray tube voltage of 50 kV, a current of 201 μA and with a 0.5-mm aluminum filter. The scanning angular rotation was 180°, and the angular increment was 0.70°. The voxel size was 4.75 μm isotropically. The images were reconstructed using the Skyscan NRecon software and analyzed using Skyscan CTAn software. The regions of interest were: (1) the total medial subchondral bone, including the bone plate and trabecular bone, in the tibial epiphysis (S1 Fig), (2) the osteophytes formed on the medial part of the tibia in the knees of the DMM-operated mice, (3) the lateral subchondral trabecular bone, and (4) the lateral subchondral bone plate. These analyses were performed in the coronal plane.

### Statistical analysis

Data are presented as scatter plots with the bar indicating the mean or expressed as mean ± standard error of the mean (SEM). Mann-Whitney U test was used for comparison of the OARSI scores (Figs 3 and 4) and student’s t- test was used for comparison of the articular cartilage thickness and osteophytes (Table 3). A two-way ANOVA was used to evaluate the effect of surgery, genotype, and interaction (surgery by genotype, used in Fig 2, Tables 1 and 2). In all cases, *P* < 0.05 was considered statistically significant. The sample size for the DMM experiment was chosen to provide at least 95% power to detect a 1.6 standard deviation difference in OA severity between *Cre-Wnt16*^*flox/flox*^ and *Wnt16*^*flox/flox*^ mice, which was considered as a biologically important difference [25, 29].

**Table 1:** Body weight, cortical area and lateral subchondral bone parameters in female mice. Two-way ANOVA was used to assess the effects of surgery (control vs. surgery destabilizing the medial meniscus [DMM]) and genotype (*Wnt16*^*flox/flox*^ vs. *Cre-Wnt16*^*flox/flox*^). The femoral cortical area was affected by genotype but not surgery, and no significant genotype by surgery interaction was detected. The lateral bone plate thickness was affected by surgery, but there was no effect of genotype or genotype by surgery interaction. Body weight, lateral trabecular thickness and number was not affected by surgery, genotype or genotype by surgery interaction. *BV/TV* bone volume/tissue volume. Data are presented as mean ± SEM, *P* < 0.05 was considered statistically significant.

	Females (N = 10–12)						
	Control		DMM		Two-way ANOVA		
	*Wnt16* ^*flox/flox*^	*Cre-Wnt16* ^*flox/flox*^	*Wnt16* ^*flox/flox*^	*Cre-Wnt16* ^*flox/flox*^	Surgery	Genotype	Interaction
Body weight at termination (g)	22.6 ± 0.9	21.8 ± 0.5	21.5 ± 0.4	21.7 ± 0.8	0.41	0.68	0.46
Cortical area (mm^2^)	0.90 ± 0.03	0.71 ± 0.05	0.87 ± 0.03	0.72 ± 0.02	0.66	< 0.001	0.49
Lateral bone plate thickness (μm)	83.1 ± 2.8	77.7 ± 1.2	72.4 ± 1.6	74.6 ± 2.2	< 0.01	0.42	0.06
Lateral BV/TV (%)	46.8 ± 1.3	45.6 ± 1.1	43.0 ± 2.5	45.2 ± 1.1	0.18	0.73	0.27
Lateral trabecular thickness (μm)	48.2 ± 1.2	50.2 ± 0.9	47.2 ± 0.9	48.6 ± 0.8	0.20	0.08	0.75
Lateral trabecular number (1/mm)	9.8 ± 0.3	9.1 ± 0.3	9.1 ± 0.5	9.3 ± 0.2	0.45	0.51	0.19

**Table 2:** Body weight, cortical area and lateral subchondral bone parameters in male mice. Two-way ANOVA was used to assess the effects of surgery (control vs. surgery destabilizing the medial meniscus [DMM]) and genotype (*Wnt16*^*flox/flox*^ vs. *Cre-Wnt16*^*flox/flox*^). The femoral cortical area was affected by genotype but not surgery, and no significant genotype by surgery interaction was detected. Body weight, lateral bone plate thickness, trabecular thickness and number was not affected by surgery, genotype or genotype by surgery interaction. *BV/TV* bone volume/tissue volume. Data are presented as mean ± SEM, *P* < 0.05 was considered statistically significant.

	Males (N = 11)						
	Control		DMM		Two-way ANOVA		
	*Wnt16* ^*flox/flox*^	*Cre-Wnt16* ^*flox/flox*^	*Wnt16* ^*flox/flox*^	*Cre-Wnt16* ^*flox/flox*^	Surgery	Genotype	Interaction
Body weight at termination (g)	28.6 ± 1.2	29.2 ± 1.1	30.2 ± 1.2	29.6 ± 1.1	0.66	0.67	0.56
Cortical area (mm^2^)	1.01 ± 0.14	0.82 ± 0.03	0.97 ± 0.04	0.84 ± 0.02	0.83	< 0.001	0.39
Lateral bone plate thickness (μm)	82.8 ± 2.2	79.1 ± 2.5	80.3 ± 2.1	82.2 ± 1.6	0.89	0.67	0.20
Lateral BV/TV (%)	46.2 ± 1.0	46.4 ± 1.0	45.5 ± 1.1	47.1 ± 1.1	0.97	0.38	0.51
Lateral trabecular thickness (μm)	41.9 ± 1.2	41.9 ± 0.9	39.9 ± 2.0	42.9 ± 1.0	0.70	0.29	0.28
Lateral trabecular number (1/mm)	11.1 ± 0.4	11.2 ± 0.4	11.8 ± 0.8	11.0 ± 0.2	0.63	0.49	0.42

## Results

### Body weight

There was no difference in weight between the *Cre-Wnt16*^*flox/flox*^ and *Wnt16*^*flox/flox*^ mice before the surgery (females *Cre-Wnt16*^*flox/flox*^ 19.2 ± 0.3 g, *Wnt16*^*flox/flox*^ 19.2 ± 0.4 g; males *Cre-Wnt16*^*flox/flox*^ 24.0 ± 0.5 g, *Wnt16*^*flox/flox*^ 23.3 ± 0.4 g). The female and male mice were allocated to the control or DMM-operated groups so that there was no weight difference within the same genotype. At the termination of the experiment, the DMM-operated mice did not differ in weight compared to the controls within the same genotype or between genotypes for the same sex (Tables 1 and 2).

### No WNT16 protein expression in articular cartilage in *Cre-Wnt16*^*flox/flox*^ mice

**Fig 1:**
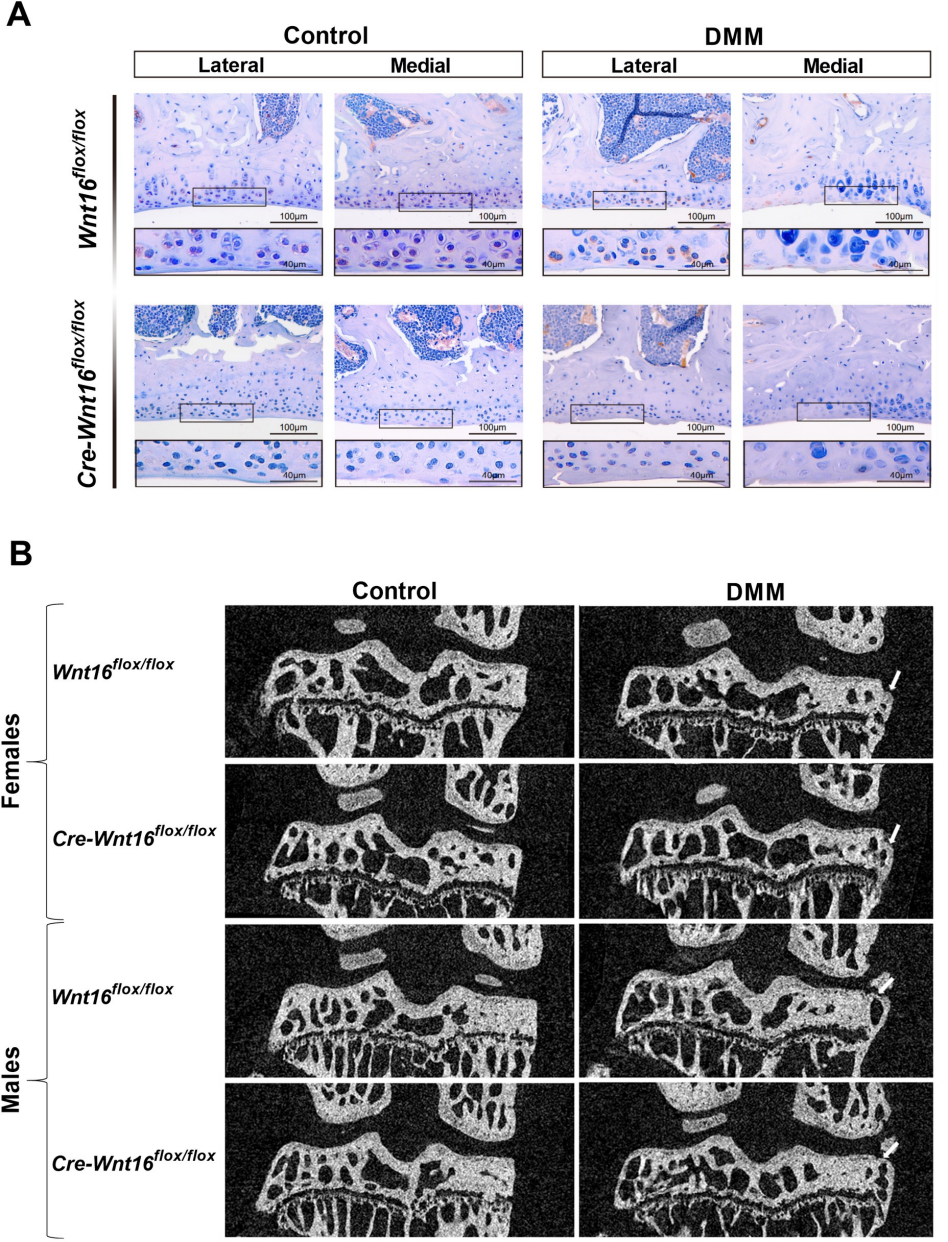
Immunohistochemistry and μCT reconstructions. (A) Representative images of immunohistochemical staining of WNT16 in the articular cartilage in tibia, showing WNT16 protein expression in Wnt16flox/flox but not Cre-Wnt16flox/flox mice. (B) The tibial subchondral bone of the Wnt16flox/flox and Cre-Wnt16flox/flox control knees (Control) and the knees that underwent DMM surgery (DMM). The arrows point at osteophytes.

Immunohistochemistry demonstrated that there was weak but detectable protein expression of WNT16 in lateral and medial articular cartilage of the tibia in both the control and DMM-operated *Wnt16*^*flox/flox*^ mice (Fig 1A). Notably, there was a higher expression of WNT16 protein on the lateral side of the DMM-operated *Wnt16*^*flox/flox*^ mice than on the medial side. There was no WNT16 protein expression in the articular cartilage in the *Cre-Wnt16*^*flox/flox*^ mice.

### Reduced cortical area in *Cre-Wnt16*^*flox/flox*^ mice

We have previously shown that the *Wnt16* mRNA levels in *Cre-Wnt16*^*flox/flox*^ mice after tamoxifen injections were reduced by 96% in the cortical bone compared to *Wnt16*^*flox/flox*^ mice, leading to reduced cortical thickness [21]. In the present study, all mice were treated with tamoxifen at 7 weeks of age to induce deletion of *Wnt16* in the *Cre-Wnt16*^*flox/flox*^ mice but not in the *Wnt16*^*flox/flox*^ mice. As expected, the cortical area was significantly reduced in the *Cre-Wnt16*^*flox/flox*^ mice compared to *Wnt16*^*flox/flox*^ mice in both sexes 9 weeks after *Wnt16* deletion (Tables 1 and 2), confirming *Wnt16* deletion in the *Cre-Wnt16*^*flox/flox*^ but not in the *Wnt16*^*flox/flox*^ mice.

### Role of *Wnt16* deletion in young adult mice in the development of experimental OA

#### Subchondral bone

**Fig 2:**
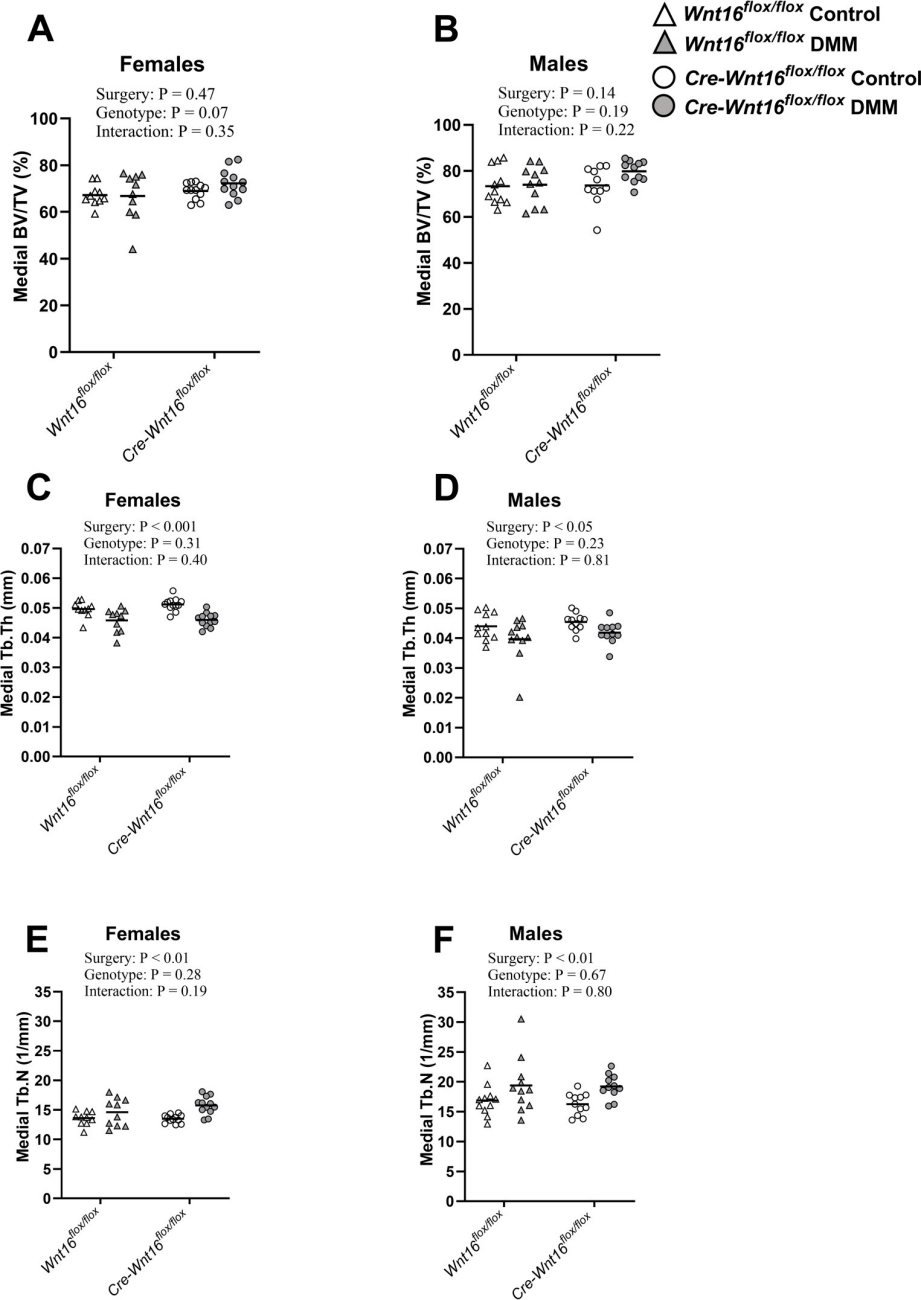
Subchondral bone. Two-way ANOVA was used to assess the effects of surgery (control vs. surgery destabilizing the medial meniscus [DMM]) and genotype (*Wnt16*^*flox/flox*^ vs. *Cre-Wnt16*^*flox/flox*^) on bone parameters. There was no effect on the total medial subchondral bone volume/tissue volume (BV/TV) by DMM surgery or genotype in female (A) or male (B) mice. In both females (C) and males (D), DMM surgery decreased the trabecular thickness (Tb.Th) but the genotype had no effect. The Trabecular number (Tb.N) was increased by DMM surgery in both female (E) and male (F) mice, but the genotype had no effect. *P* < 0.05 was considered statistically significant. (Females N = 10–12; males N = 11).

Tamoxifen injections result in a high bone volume and for this reason, all the medial subchondral bone except the osteophytes (medial subchondral bone plate + medial subchondral trabecular bone ‒ osteophyte) was analyzed together (Figs 1B and S1). Neither the genotype nor the surgery affected the BV/TV for the total medial subchondral bone in females or males (Fig 2A and 2B). However, the DMM surgery significantly decreased the trabecular thickness (Tb.Th) and increased the trabecular number (Tb.N) in both females and males (Fig 2C–2F). The genotype did not affect the Tb.Th or Tb.N and there were no significant genotype by surgery interactions for BV/TV, Tb.Th or Tb.N in either sex (Fig 2A–2F). In the lateral part of the subchondral bone, the bone plate could be separated from the subchondral trabecular bone (S1 Fig). The lateral bone plate thickness was not affected by genotype in females and males, but DMM surgery resulted in a decreased bone plate thickness in females but not in males (Tables 1 and 2). Lateral subchondral trabecular BV/TV, Tb.Th or Tb.N were not affected by either genotype or surgery in either females or males and there were no significant genotype by surgery interactions for lateral bone plate thickness, BV/TV, Tb.Th or Tb.N in either sex (Tables 1 and 2).

Osteophytes on the medial side of the tibia were only formed in mice that had DMM surgery (Figs 1B and S1). The osteophyte volume was not affected by genotype and was similar in the medial tibiae of the *Cre-Wnt16*^*flox/flox*^ and *Wnt16*^*flox/flox*^ mice subjected to DMM surgery in both female and male mice (Table 3).

**Table 3:** Osteophyte volume in the medial tibia of DMM-operated knees and thickness of medial articular cartilage in tibia of control knees. There was no difference in osteophyte volume or in total articular cartilage thickness between the *Cre-Wnt16*^*flox/flox*^ and *Wnt16*^*flox/flox*^ mice in either females or males (N = 10–12). Data are presented as mean ± SEM. Student’s *t* test, *Cre-Wnt16*^*flox/flox*^ vs. *Wnt16*^*flox/flox*^. *P* < 0.05 was considered statistically significant.

	Females		Males	
	*Wnt16* ^*flox/flox*^	*Cre-Wnt16* ^*flox/flox*^	*Wnt16* ^*flox/flox*^	*Cre-Wnt16* ^*flox/flox*^
Osteophyte volume (mm^3^)	0.073 ± 0.008	0.083 ± 0.009	0.137± 0.011	0.153 ± 0.015
Total articular cartilage thickness (μm)	151 ± 3	150 ± 5	155 ± 3	161 ± 6

#### Articular cartilage

There was no difference in the total articular cartilage thickness between the control *Cre-Wnt16*^*flox/flox*^ and control *Wnt16*^*flox/flox*^ mice, neither in males nor in females (Table 3).

**Fig 3:**
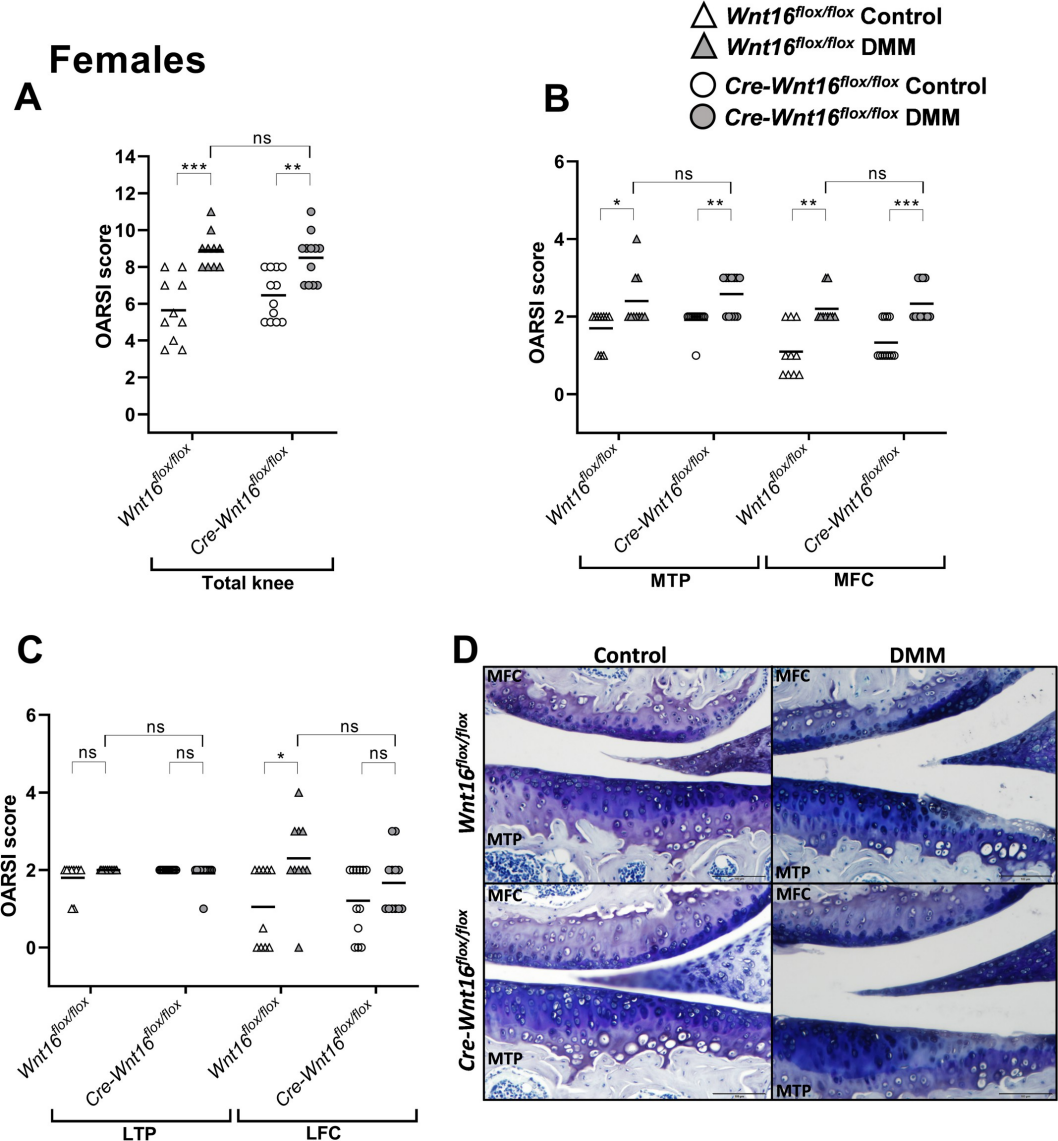
OARSI scores for control knees and knees that underwent DMM surgery in female mice. (A) The summed OARSI scores for the total knee (total knee = medial tibial plateau [MTP] + medial femoral condyle [MFC] + lateral tibial plateau [LTP] + lateral femoral condyle [LFC]), where the maximum score for the total knee is 24. (B) The OARSI score for the MTP and the MFC. The maximum score for each region is 6. (C) The OARSI score in the LTP and LFC. The maximum score for each region is 6. (D) Representative microphotographs of the MTP and MFC in control and DMM-operated knees in *Wnt16*^*flox/flox*^ and *Cre-Wnt16*^*flox/flox*^ mice. Especially the MTP in the DMM-operated knees show cartilage damage in both the *Wnt16*^*flox/flox*^ and *Cre-Wnt16*^*flox/flox*^ female mice. Magnification × 20 and the black bar in the photos represents 100 μm. Data are shown as scatter plots where the bars show the mean. *P* < 0.05 was considered statistically significant, *P < 0.05, **P < 0.01, ***P < 0.001, the Mann-Whitney U test was used (N = 10/group for the *Wnt16*^*flox/flox*^ and N = 12/group for the *Cre-Wnt16*^*flox/flox*^ mice).

**Fig 4:**
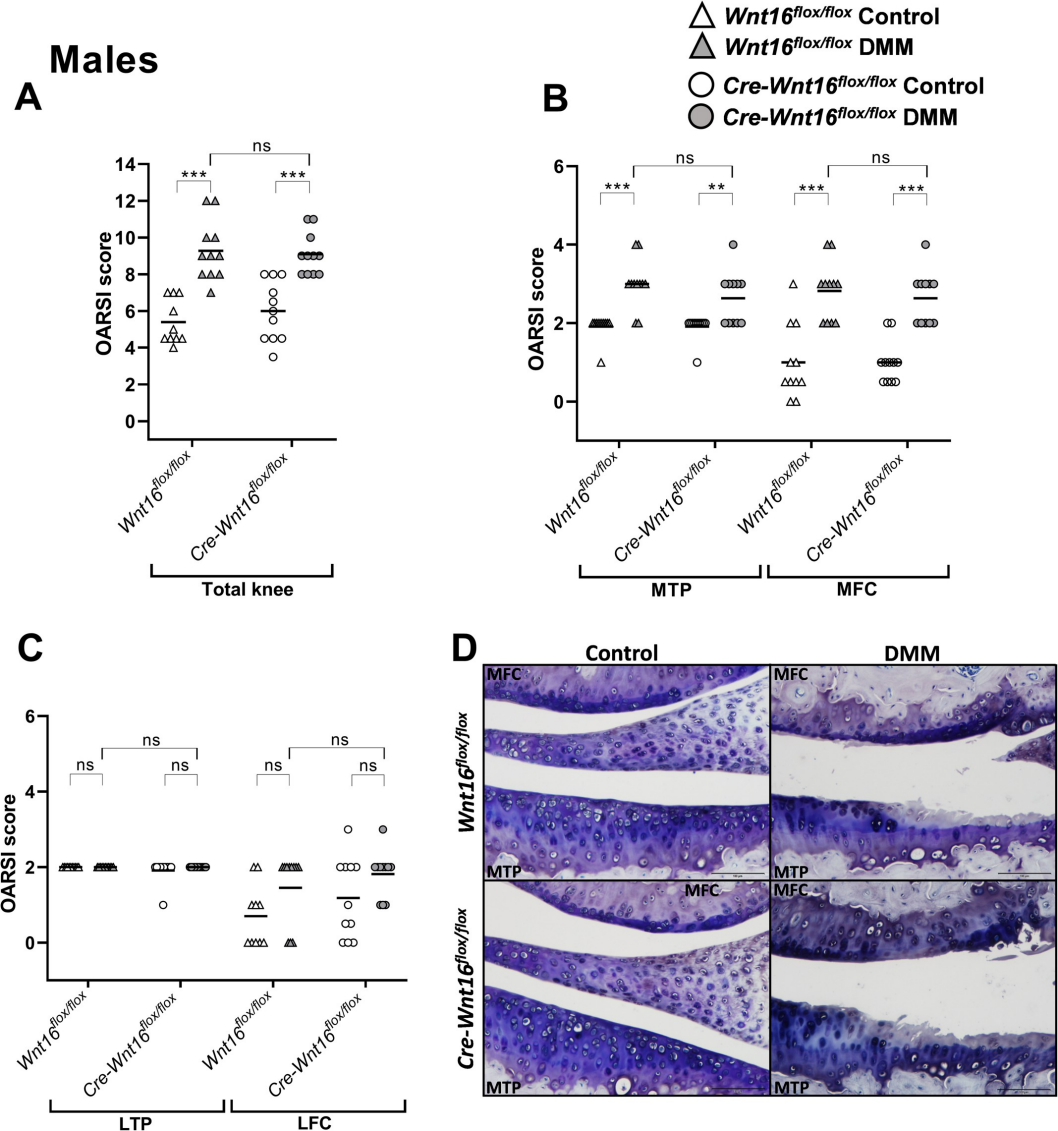
OARSI scores for control knees and knees that underwent DMM surgery in male mice. (A) The summed OARSI scores for the total knee (total knee = medial tibial plateau [MTP] + medial femoral condyle [MFC] + lateral tibial plateau [LTP] + lateral femoral condyle [LFC]), where the maximum score for the total knee is 24. (B) The OARSI score for the MTP and the MFC. The maximum score for each region is 6. (C) The OARSI score in the LTP and LFC. The maximum score for each region is 6. (D) Representative microphotographs of the MTP and MFC in control and DMM-operated knees in *Wnt16*^*flox/flox*^ and *Cre-Wnt16*^*flox/flox*^ mice. Especially the MTP in the DMM-operated knees show cartilage damage in both the *Wnt16*^*flox/flox*^ and *Cre-Wnt16*^*flox/flox*^ female mice. Magnification × 20 and the black bar in the photos represents 100 μm. Data are shown as scatter plots where the bars show the mean. *P* < 0.05 was considered statistically significant, *P < 0.05, **P < 0.01, ***P < 0.001, the Mann-Whitney U test was used (N = 10/group for the LFC and total lateral score in the control *Wnt16*^*flox/flox*^ mice, N = 11/group for the medial scores in the control *Wnt16*^*flox/flox*^ mice, N = 11/group for the DMM-operated *Wnt16*^*flox/flox*^ mice, and the *Cre-Wnt16*^*flox/flox*^ mice).

The summed maximal total OARSI scores (MTP + MFC + LTP + LFC) were significantly higher in the DMM-operated knees compared to the control knees in both *Cre-Wnt16*^*flox/flox*^ and *Wnt16*^*flox/flox*^ female and male mice (Figs 3A and 4A). When analyzing the MTP and MFC, the maximal OARSI score was significantly higher after DMM surgery compared to control knees in female and male *Cre-Wnt16*^*flox/flox*^ and *Wnt16*^*flox/flox*^ mice (Figs 3B, 3D, 4B and 4D). There was no difference between the *Cre-Wnt16*^*flox/flox*^ and *Wnt16*^*flox/flox*^ DMM-operated knees for either males or females in the total joint or in the medial compartment (Figs 3A, 3B, 4A and 4B). The only effect on the lateral side after DMM was seen in the LFC in female mice (Fig 3C). However, no difference between the DMM-operated *Wnt16*^*flox/flox*^ mice and DMM-operated *Cre-Wnt16*^*flox/flox*^ mice was seen in the lateral compartment in either female or male mice (Figs 3C and 4C).

## Discussion

WNT16 has previously been suggested to protect articular cartilage during experimental OA development in mice. This was shown in mice with either a global (*Wnt16*^*-/-*^) or chondrocyte specific deletion of *Wnt16*, since these mice developed a more severe experimental OA than control mice [15, 16, 25]. However, these mice have never expressed WNT16, and therefore it has not been possible to elucidate whether expression of WNT16 is needed when experimental OA is induced or whether expression of WNT16 in young animals may possibly protect against experimentally induced OA later in life. Herein, we used an inducible *Wnt16* inactivated mouse model and demonstrated that inactivation of *Wnt16* in young adult mice has no impact on the progression of experimental OA. This suggests that *Wnt16* expression in adult mice is not important for protecting cartilage during experimental OA progression, if the mice have had normal WNT16 expression during development.

Previous studies, using Rosa26R reporter mice [30], have shown that tamoxifen can penetrate articular cartilage and induce *Cre*-mediated recombination in adult mice. This was shown by staining the cartilage with X-gal to detect β-galactosidase activity at day three or five after the last tamoxifen injection [31, 32]. WNT16 is expressed in cortical bone and articular cartilage [14, 16] and herein, we show with immunohistochemistry, eight weeks after DMM surgery, that WNT16 protein is present in the articular cartilage of the tibia in *Wnt16*^*flox/flox*^ mice but not in the *Cre-Wnt16*^*flox/flox*^ mice after tamoxifen-induced deletion of the *Wnt16* gene. Comparing our results with the findings from the Rosa26R reporter mice [31, 32], we believe it is reasonable to claim that *Wnt16* has been knocked out of the cartilage in our model at the time of DMM surgery, which is performed four to five days after the last tamoxifen injection.

In addition, we have previously shown that *Cre-Wnt16*^*flox/flox*^ mice treated with the tamoxifen dose used in this study (50 mg/kg), have a 96% reduction of the *Wnt16* mRNA levels in cortical bone four weeks after tamoxifen injections as compared with the tamoxifen treated *Wnt16*^*flox/flox*^ mice, leading to reduced cortical bone mass [21]. In the present study, we show that the cortical area was significantly reduced in the *Cre-Wnt16*^*flox/flox*^ mice compared with the *Wnt16*^*flox/flox*^ mice. Altogether, these findings demonstrate that the tamoxifen treatment successfully induced *Wnt16* deletion both in the articular cartilage and cortical bone in the *Cre-Wnt16*^*flox/flox*^ mice in this study.

Tamoxifen is a selective estrogen receptor modulator (SERM) that is known to affect the skeleton and increase the trabecular BV/TV [33, 34]. Longitudinal epidemiological studies have found evidence for associations between high BMD and incidence of OA, and we have recently shown that high femoral neck BMD is causal for knee OA and hip OA, but not hand OA [35]. However, most studies find no association between high BMD and OA progression [36–40] and in a previous study we have demonstrated that high bone mass in mice, per se, does not lead to a more serious OA phenotype after DMM surgery [25]. In addition, tamoxifen treatment of rabbits during the development of experimental OA leads to less cartilage degradation [41]. To avoid any possible confounding effects of tamoxifen on the skeleton and cartilage, both *Wnt16*^*flox/flox*^ mice and *Cre-Wnt16*^*flox/flox*^ received the same tamoxifen dose, resulting in comparable BV/TV, Tb.Th and Tb.N in the lateral and total medial subchondral bone between genotypes. The BV/TV of the total medial subchondral bone was not increased by DMM surgery in the female or male mice, most likely due to the already relatively high subchondral bone mass caused by the tamoxifen injections. In the female but not the male mice, the lateral bone plate was thinner in the mice that had DMM surgery. This can sometimes be seen in mice undergoing DMM surgery and we have previously seen this in WT mice [42]. This is probably due to an increased load on the medial side due to the destabilization of the medial meniscus which leads to a decreased load on the lateral side rendering a thinner bone plate.

In the present study, when experimental OA severity in the articular cartilage was evaluated eight weeks after DMM surgery, the OARSI scores in the *Cre-Wnt16*^*flox/flox*^ mice were similar to the *Wnt16*^*flox/flox*^ mice, which have a normal *Wnt16* expression. This is in contrast to the studies by *Nalesso et al* (2016, *Wnt16*^*-/-*^ [15]) and *Tong et al* (2019, *Wnt16-cKO* [16]), where the mice with global or chondrocyte specific *Wnt16* deletion showed a more severe experimental OA development after DMM or ACLT surgery compared to controls. *Nalesso et al* found significant differences in OARSI scores between *Wnt16*^*-/-*^ and control mice eight weeks after DMM surgery. In the present study, the experiment was also terminated eight weeks after the DMM surgery. Thus, it is reasonable to believe that we would have been able to detect a difference in OARSI scores between the *Cre-Wnt16*^*flox/flox*^ and the *Wnt16*^*flox/flox*^ mice, if it existed. In our study, the mice were injected with tamoxifen to induce *Wnt16* deletion in the *Cre-Wnt16*^*flox/flox*^ when they were 7 weeks old, one week prior to the DMM surgery. This means that the *Cre-Wnt16*^*flox/flox*^ mice in our study expressed *Wnt16* through the synovial joint establishment during embryonic development and until they were young adults [19, 20]. We propose that the discrepancies in OA progression between the *Cre-Wnt16*^*flox/flox*^ mice in our study and the *Wnt16*^*-/-*^ and chondrocyte specific *Wnt16-KO* mice could be due to that WNT16 is crucial during the development of the synovial joints. Although the joints seem normal in the *Wnt16*^*-/-*^ and the chondrocyte specific *Wnt16-KO* mice, the cartilage may be more susceptible to damage when challenged with surgery to induce experimental OA. In addition, the thickness of the total articular cartilage was thinner in the chondrocyte specific *Wnt16-KO* mice compared to the WT mice at 1 year but not at 12 and 24 weeks of age [16].

Furthermore, after the tamoxifen injections, we have previously shown that there is a 96% reduction of *Wnt16* mRNA in the cortical bone of the *Cre-Wnt16*^*flox/flox*^ mice [21]. Hence, there is still a very small amount of *Wnt16* mRNA transcribed. Although we were not able to detect WNT16 protein in the articular cartilage by immunohistochemistry in the *Cre-Wnt16*^*flox/flox*^ mice, we did not measure *Wnt16* mRNA. Therefore, we cannot completely exclude that a small amount of *Wnt16* is still expressed in *Cre-Wnt16*^*flox/flox*^ mice after the tamoxifen injections and that this could be sufficient to reduce the damage in articular cartilage after DMM surgery in a similar manner as in the *Wnt16*^*flox/flox*^ mice.

Another possibility as to why the *Cre-Wnt16*^*flox/flox*^ mice in this study do not have a more severe OA than the *Wnt16*^*flox/flox*^ mice could be that the importance of *Wnt16* expression for cartilage protection is strain dependent. The *Cre-Wnt16*^*flox/flox*^ mice are on a C57/BL6 genetic background whereas the *Wnt16*^*-/-*^ mice used by *Nalesso et al* are on a 129/Sv genetic background [15]. A previous study has demonstrated that mice on a129/Sv genetic background develop a more severe OA phenotype after DMM surgery than mice on a C57/BL6 genetic background [43]. It is therefore possible that the C57/BL6 genetic background of the mice in the present study protects the mice from developing an even more severe OA phenotype. This is in contrast to the cortical bone phenotype of total *Wnt16*^*-/-*^ mice, where *Wnt16*^*-/-*^ mice on C57/BL6 genetic background develop spontaneous fractures, which is not found in *Wnt16*^*-/-*^ mice on 129/Sv genetic background [14, 44]. Herein, we have shown that the *Cre-Wnt16*^*flox/flox*^ mice have a reduced cortical area even though they express *Wnt16* during development and only lack *Wnt16* expression for nine weeks, which supports previous findings that *Wnt16* is crucial for adult cortical bone metabolism [21]. However, *Wnt16* expression in adult mice is not crucial for protecting the cartilage after inducing experimental OA by DMM surgery.

We here demonstrate presence of WNT16 protein in the articular cartilage in both DMM-operated and un-operated *Wnt16*^*flox/flox*^ control mice 8 weeks after surgery at 16 weeks of age. This is in accordance with *Tong et al* that have shown that WNT16 protein can be detected by immunohistochemistry in the knee joint in intact WT mice at the ages of 12 weeks, 24 weeks and 1 year [16]. In contrast, previous studies by *Nalesso et al* could not detect WNT16 protein in healthy adult articular cartilage in the knee joint in intact WT mice. They could only detect WNT16 protein by immunohistochemistry at 2 and 7 days after DMM or sham surgery, but after 8 weeks no WNT16 protein could be detected [15]. The discrepancies between the studies may be that the primary antibodies used for immunohistochemistry were different or that *Wnt16* expression differ between strains of mice. In the present study, the un-operated control mice had some small damages to the articular cartilage which could have led to an upregulation of *Wnt16*. However, there are still some uncertainties as to when and where *Wnt16* is expressed in the articular cartilage in mice.

Previous studies have shown that if an adenoviral vector overexpressing *Wnt16* is injected into either a WT or chondrocyte specific *Wnt16-KO* mouse knee with experimentally induced OA, the OA phenotype was attenuated [16]. Thus, WT mice with a normal *Wnt16* expression have an attenuated OA phenotype when treated with WNT16. It was not within the scope of this study to treat the mice with an adenoviral vector overexpressing *Wnt16*. However, we speculate that adding a high dose of WNT16 into the knee joint during ongoing OA progression is protecting the cartilage and attenuating the OA phenotype but the endogenous expression of *Wnt16* by chondrocytes, or other cell types, in the mouse does not necessarily have the same effect.

There are a few limitations in this study. We induced experimental OA by DMM which is more closely related to post-traumatic OA in humans than the more common non-traumatic OA, which is a result of natural wear and tear. However, the DMM model is an easy model to study since usually all mice undergoing DMM develop a rapidly progressing OA and the results are reproducible [45]. The study only includes a single time point and at the time of the experiment we were not able to study pain in the mice, which is an important parameter in studying OA. Due to lack of tissue, we could not quantify the differences of *Wnt16* expression in the articular cartilage in this study, which would have added information.

In conclusion, *Wnt16* expression in adult mice is not crucial for attenuating experimental OA, if the mice have had a normal expression of *Wnt16* during development. However, this does not exclude the possibility that treating the knee joint with WNT16 could ameliorate the progression of OA.

## Supporting information

S1 Fig Image in the coronal plane of a right DMM-operated tibia from the μCT. (TIF)
