# Supplementary figures and images for: Induced inactivation of Wnt16 in young adult mice has no impact on osteoarthritis development
Source: PLoS One. 2022 Nov 11;17(11):e0277495. doi: 10.1371/journal.pone.0277495 (PMC9651579; doi:10.1371/journal.pone.0277495)

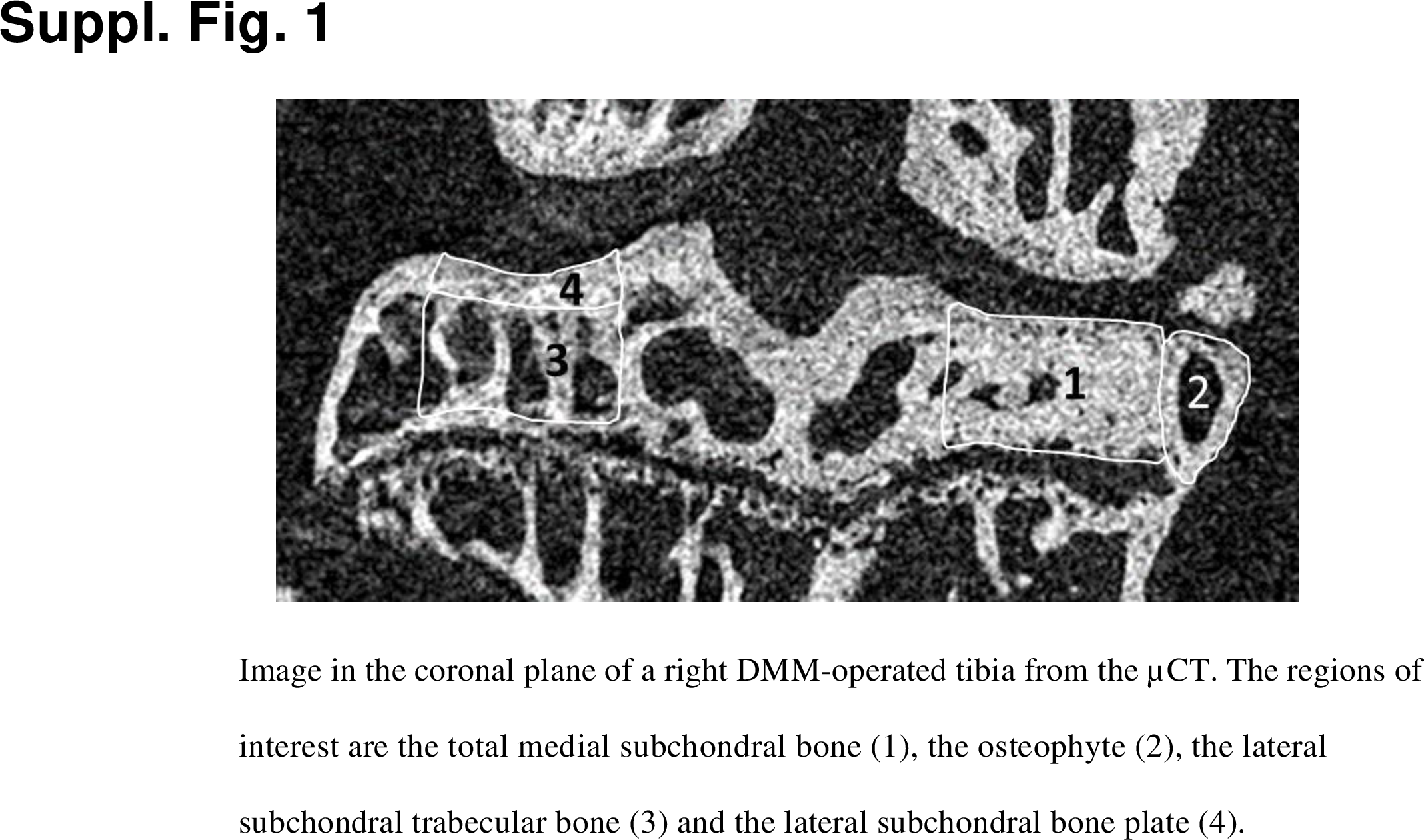

Supplement: S1 Fig — (TIF) [file pone.0277495.s001.tif]
